# Maternal risk factors and perinatal outcomes among pacific islander groups in Hawaii: a retrospective cohort study using statewide hospital data

**DOI:** 10.1186/s12884-015-0671-4

**Published:** 2015-10-05

**Authors:** Ann Lee Chang, Eric Hurwitz, Jill Miyamura, Bliss Kaneshiro, Tetine Sentell

**Affiliations:** 1 Department of Obstetrics, Gynecology and Women’s Health, University of Hawaii, 1319 Punahou Street Suite #824, Honolulu, HI 96826 USA; 2 Office of Public Health Studies, University of Hawaii, 1960 East–west Road, Biomed D-204, Honolulu, HI 96822 USA; 3 Hawaii Health Information Corporation, 733 Bishop Street, Suite 1870, Honolulu, HI 96813 USA

**Keywords:** Pregnancy outcomes, Pacific Islander Americans, Hawaii

## Abstract

**Background:**

Studies suggest Pacific Islander women have disparate rates of preterm birth, primary cesarean delivery, preeclampsia, gestational diabetes, and low birthweight infants. However, data is limited. In order to improve the health of Pacific Islanders, it is essential to better understand differences in obstetric outcomes in this diverse population

**Methods:**

This study compared perinatal outcomes between Pacific Islander (9,646) and White (n = 5,510) women who delivered a singleton liveborn in any Hawaii hospital from January 2010 to December 2011 using the Hawaii Health Information Corporation (HHIC) database. Pacific Islanders were disaggregated into the following groups: Native Hawaiian, Samoan, Micronesian, and Other Pacific Islanders. Perinatal outcomes (e.g. hypertensive diseases, birthweight, mode of delivery) were compared using multivariable logistic models controlling for relevant sociodemographic and health risk factors (e.g. age and payer type).

**Results:**

Significant differences in perinatal outcomes between Pacific Islander and White women and newborns were noted. All Pacific Islander groups had an increased risk of hypertension. Outcome differences were also seen between Pacific Islanders groups. Native Hawaiians had the highest risk of low birthweight infants, Samoans had the highest risk of macrosomic infants and Micronesians had the highest risk of cesarean delivery.

**Conclusions:**

Important differences in perinatal outcomes among Pacific Islanders exist. It is important to examine Pacific Islander populations separately in future research, public health interventions, and policy.

**Electronic supplementary material:**

The online version of this article (doi:10.1186/s12884-015-0671-4) contains supplementary material, which is available to authorized users.

## Background

Pacific Islanders, including Native Hawaiians, Samoans, and Micronesians, comprise an important and rapidly increasing proportion of the United States (US) population [1]. These groups are adversely affected by socioeconomic disparities that are known to result in a higher risk of adverse obstetric outcomes [2–4]. Indeed, studies in both the US and New Zealand suggest that Pacific Islander women have disparate rates of preterm birth, primary cesarean delivery, preeclampsia, gestational diabetes, and low birthweight, but data in these groups is extremely limited [5–13]. Most studies on this topic aggregate Asian and Pacific Islander women into a single large group [5–7]. The few studies that disaggregate Pacific Islanders from Asian subgroups did not explore Pacific Islander subgroups separately; [8, 12] focused on only one hospital in one location; [8, 13] or only examined a few outcomes [9, 10, 12–15]. In order to improve the health of Pacific Islanders, it is essential to understand differences in obstetric outcomes in Pacific Islander subgroups residing in the United States.

The goal of this study was to quantify obstetric outcomes across Pacific Islander subgroups residing in Hawaii. According to the 2010 US Census, Hawaii has highest proportion of residents who report being Native Hawaiians or Pacific Islanders; 23 % of all Pacific Islanders in the US live in Hawaii [1]. We selected common outcomes with well-established morbidity including diabetes, hypertension, cesarean delivery, and birthweight. [14–20]

## Methods

### Sample

The Hawaii Health Information Corporation (HHIC) inpatient database was used to conduct this study. The HHIC Mothers and Babies Database includes all maternal delivery and live newborn hospital admissions in the state of Hawaii. Demographic information (such as race and payer type), up to twenty International Classification of Diseases, 9th Revision (ICD-9) diagnosis codes per patient, and up to twenty ICD-9 procedure codes per patient are included in the database. The HHIC is Hawaii’s healthcare data organization participating with the Agency for Healthcare Research and Quality (AHRQ) Healthcare Cost and Utilization Project, which is a Federal-State-Industry partnership sponsored by AHRQ [21, 22]. We received permission from HHIC to use their Mothers and Babies Database for the purpose of this study.

Our study population consisted of all Pacific Islander and White women who delivered a singleton liveborn in any Hawaii hospital from January 2010 to December 2011 and their newborns. All non-federal acute care hospitals in Hawaii were included. Women with ICD-9 codes corresponding to multiple gestations were excluded. There were 36,683 maternal delivery admissions for singleton liveborns in Hawaii during time period studied; 5,510 persons were White and 9,646 were Pacific Islanders. Thus, the study sample was 15,156.

All data were de-identified and the analysis was deemed exempt by the Institutional Review Board of the University of Hawaii under federal exemption category 4.

### Variables

Race was based on self-report by each patient at time of admission. Thirteen of the 34 possible races represented in the database are part of the Pacific Islander category. Women were classified as based on the most commonly reported Pacific Islander races in our population: Native Hawaiian (Native Hawaiian, Hawaiian, and Part Native Hawaiian); Micronesian (Marshallese, Other Micronesian, Guamanian or Chamorro); Samoan; and Other Pacific Islander (all other Pacific Islander categories). Those classified as Whites were used as the comparison group.

Other maternal demographic data obtained from the database included age (in years), payer type, and admission to a rural hospital versus urban hospital. These sociodemographic risk factors were chosen given their association with disparate obstetric outcomes [3, 7, 23–30]. Payer type was separated into private versus public/uninsured (Medicaid, Medicare, and uninsured). The uninsured subjects were combined with the Medicaid and Medicare subjects as both populations are similar in economic status; per the Kaiser Family Foundation, 74 % of the uninsured nonelderly in Hawaii are eligible for either Medicaid or via the Marketplaces; 9 % are immigrants that are not eligible for government-funded insurance due do their immigration status [31].

Perinatal outcomes were obtained by extracting ICD-9 codes from each admission. Variables included hypertension (pre-existing and pregnancy-associated hypertension), diabetes mellitus (pre-gestational and gestational), and mode of delivery (vaginal or cesarean delivery). Additional file 1 contains the ICD-9 codes used to identify these diagnoses. We chose the ICD-9 codes for these outcomes based on a prior validation study by Yasmeen et al. [32] Although their sensitivity for cesarean delivery was 100 %, we also included the newborn ICD-9 codes of v3xx.1 (newborn born via cesarean); by doing so we identified 34 additional women who had a cesarean. Yasmeen et al. found that the sensitivity for the ICD-9 codes for pre-gestational diabetes and gestational diabetes were 75 and 64 %, respectively, and the codes for chronic hypertension and pregnancy-related hypertension were 58 and 88 %, respectively. However, by combining pre-gestational and gestational diabetes into a broader category of diabetes and doing the same for hypertensive diseases, the sensitivities should be more reliable [33].

Newborns were separated into categories of low birthweight (<2500 g), normal weight (2500–4000 g) and macrosomic (>4000 g).

### Statistical analyses

Data were analyzed using JMP 11.1.1 [34] and SPSS version 21 [35]. Obstetric outcomes and demographics were compared among Pacific Islander subgroups and with Whites. Whites were used as the reference group because of the common use of this group in examining racial disparities. The study population was first summarized using descriptive statistics. Chi square tests were used to calculate p values. Multivariable logistic regression models were then created to explore the association between racial group and obstetric outcome. Potential confounders such as maternal demographics and risk factors were included in the model. Demographic variables included in all models were: age, race, delivery at a rural versus urban hospital, and payer type. Other specific variables in the multivariable logistic models varied by outcome. Variables were chosen because of their known associations with the examined outcomes based on previous published research and/or because multivariate models from other comparison studies have included them [7–20, 23–30, 36–39]. Adjusted odds ratios (aORs) and 95 % confidence intervals (CIs) were obtained for each outcome using these models. Statistical significance was set at *P* < 0.05.

## Results

Differences were seen in all demographic factors and perinatal outcomes across Pacific Islander subgroups and Whites (Table 1). Teen births were more common in all Pacific Islander subgroups compared to Whites. The percentage of deliveries by women of advanced maternal age was highest in Whites. The proportion of women who delivered in a rural versus an urban hospital was significantly different by maternal race. More than 40 % of Whites compared to 5.9 % of Samoans delivered in a rural hospital. Approximately one-third of Whites (31.7 %) had public insurance or no insurance whereas more than 50 % of Pacific Islanders had this insurance type, with the highest percentage being 88.3 % for Micronesians.

**Table 1:** Maternal demographics and outcomes among singleton births, Hawaii, 2010–2011 by race/ethnicity. (HHIC Inpatient Data, 2010–2011)

	Native Hawaiian	Micronesian	Samoan	Other Pacific Islanders	White	p-value
n	6662	1548	897	539	5510	
Demographics						
Delivery in rural hospital (%)	2621 (39.3)	478 (30.9)	53 (5.9)	53 (28.9)	2254 (40.9)	<.0001
Public insurance/uninsured (%)	3658 (54.9)	1367 (88.3)	594 (66.2)	324 (60.1)	1746 (31.7)	<.0001
Mean age (years) (SD)	26.4 (6.1)	26.4 (5.5)	26.3 (5.9)	27.6 (6.3)	29.4 (6.0)	<.0001
Age Groups						
<20 years (%)	846 (12.7)	127 (8.2)	106 (11.8)	44 (8.2)	250 (4.5)	<.0001
>35 years (%)	724 (10.9)	140 (9.0)	91 (10.1)	84 (15.6)	1100 (20.0)	<.0001
Outcomes						
Diabetes (%)	621 (9.3)	132 (8.5)	113 (12.6)	74 (13.7)	319 (5.8)	<.0001
Hypertension (%)	792 (11.9)	145 (9.4)	134 (14.9)	64 (11.9)	413 (7.5)	<.0001
Mode of Delivery						
Cesarean delivery (%)	1671 (25.1)	500 (32.3)	189 (21.1)	163 (30.2)	1474 (26.8)	<.0001
Primary cesarean (%)	808 (12.1)	273 (17.6)	90 (10.0)	83 (15.4)	871 (15.8)	<.0001
Vaginal delivery (%)	4991 (74.9)	1048 (67.7)	708 (78.9)	376 (69.8)	4036 (73.3)	<.0001
Birthweight						
Mean birthweight in grams (SD)	3264 (586)	3219 (550)	3477 (618)	3372 (609)	3368 (543)	<.0001
<2500 grams (%)	489 (7.3)	106 (6.9)	37 (4.1)	38 (7.1)	266 (4.8)	<.0001
>4000 grams (%)	504 (7.6)	91 (5.9)	140 (15.6)	62 (11.5)	540 (9.8)	<.0001

Whites had the lowest prevalence of diabetes and hypertension. Diabetes was most prevalent among Other Pacific Islanders (13.7 %) and Samoans (12.6 %). Samoans (14.9 %) and Native Hawaiians (11.9 %) had the highest rates of hypertension.

Delivery mode and birthweight varied across all groups studied. Cesarean section rates were highest for Micronesians (32.3 %) and lowest for Samoans (21.1 %). Samoans also had babies with the highest mean birthweight, the highest rates of macrosomia (birthweight greater than 4000 g), and the lowest rates of low birthweight (<2500 g). Low birthweight was highest among Native Hawaiians and Other Pacific Islanders, 7.3 and 7.1 % respectively, compared to 4.1 % for Samoans.

### Multivariable analyses

Results of the multivariate regression analyses with Whites as the comparison group are shown in Table 2.

**Table 2:** Unadjusted and adjusted odds ratios (95 % confidence intervals) for perinatal outcomes of Pacific Islander subgroups compared to Whites (HHIC Inpatient Data, 2010–2011)

	Diabetes	Hypertension	Low birthweight <2500 grams	Macrosomia >4000 grams	Cesarean delivery
	OR (95 % CI)	OR (95 % CI)	OR (95 % CI)	OR (95 % CI)	OR (95 % CI)
Native Hawaiian					
Unadjusted OR	1.68 (1.46–1.93)	1.60 (1.42–1.81)	1.56 (1.34–1.82)	0.75 (0.66–0.86)	0.91 (0.85–0.99)
Adjusted OR	2.22 (1.92–2.57)	1.62 (1.42–1.84)	1.16 (1.01–1.34)	0.84 (0.74–0.96)	0.93 (0.85–1.01)
Micronesian					
Unadjusted OR	1.52 (1.24–1.87)	1.21 (0.99–1.47)	1.45 (1.15–1.83)	0.57 (0.46–0.72)	1.31 (1.16–1.48)
Adjusted OR	1.98 (1.58–2.49)	1.09 (0.88–1.34)	1.08 (0.87–1.35)	0.67 (0.53–0.86)	1.35 (1.19–1.54)
Samoan					
Unadjusted OR	2.30 (1.84–2.88)	2.00 (1.62–2.47)	0.85 (0.60–1.21)	1.70 (1.39–2.08)	0.73 (0.61–0.87)
Adjusted OR	2.63 (2.06–3.35)	1.63 (1.31–2.04)	0.55 (0.40–0.76)	1.87 (1.51–2.32)	0.92 (0.77–1.09)
Other Pacific Islander					
Unadjusted OR	2.49 (1.91–3.25)	1.71 (1.31–2.23)	1.50 (1.05–2.13)	1.19 (0.90–1.58)	1.87 (0.98–1.44)
Adjusted OR	2.82 (2.13–3.72)	1.50 (1.14–1.98)	1.20 (0.88–1.64)	1.23 (0.93–1.64)	1.31 (1.08–1.59)
Whites	1.00	1.00	1.00	1.00	1.00

Adjusted for age, rural, insurance

In addition

-Diabetes: already adjusted for hypertension

-Hypertension: also adjusted for diabetes

-Low birthweight and macrosomia: also adjusted for diabetes and hypertension

-Cesarean delivery: also adjusted for diabetes, hypertension, birthweight

All Pacific Islander subgroups had significantly higher (at least two-fold) adjusted odds ratios for diabetes compared to Whites. Similarly, all Pacific Islander subgroups other than Micronesians had significantly higher adjusted odds ratios for hypertension compared to Whites; Samoans and Native Hawaiians had the highest adjusted odds ratios (aOR 1.63; 95 % CI 1.31–2.04 and aOR 1.62; 95 % CI 1.42–1.84, respectively).

Native Hawaiians had the highest, statistically significant adjusted odds ratio (aOR 1.16; 95 % CI 1.01–1.34) for low birthweight babies. Samoans, on the other hand, were significantly less likely to have low birthweight infants than Whites (aOR 0.55; 95 % CI 0.40–0.76). Samoans were also more likely than Whites to have macrosomic infants (aOR 1.87; 95 % CI 1.51–2.32), while Native Hawaiians and Micronesians were significantly less likely to have macrosomic infants compared to Whites (aOR 0.84; 95 % CI 0.74–0.96 and aOR 0.67; 95 % CI 0.53–0.83, respectively).

Compared to Whites, Micronesians and Other Pacific Islanders were more likely to have cesarean deliveries (aOR 1.35; 95 % CI 1.19–1.454 and aOR 1.31; 95 % CI 1.08–1.59, respectively). Samoans and Native Hawaiians were less likely than Whites to have a cesarean in the unadjusted model but these findings were not significant in the adjusted model.

## Discussion

This study revealed that, compared to Whites, Pacific Islanders in Hawaii generally had more socioeconomic and demographic risk factors such as teen birth and having public or no insurance. These factors are known predictors of adverse obstetric outcomes [25–28]. Thus it is not surprising that all Pacific Islander subgroups studied were more likely to have diabetic disease and all Pacific Islander groups except for Micronesians were more likely to have hypertensive disorders.

However, Pacific Islanders also showed considerable variation between subgroups in perinatal outcomes when examining birthweight and cesarean delivery. Whites did not have better outcomes than all the Pacific Islander groups studied, nor did one Pacific Islander subgroup fare better or worse than the other subgroups in these outcomes. For example, when compared to Whites, Samoans were less likely to have low birthweight infants whereas Native Hawaiians were more likely to have low birthweight infants. Regarding mode of delivery, Micronesians were more likely to have a Cesarean delivery while the other Pacific Island groups were not at increased or decreased risk for Cesarean delivery compared to Whites. Many of these variations have not been shown in previous research due to limited sample sizes of disaggregated Pacific Islander groups.

Our study confirmed some findings that were seen in previous studies, such as the high teen birth rates of Pacific Islanders [40]. The higher adjusted odds ratio for low birthweight infants among Native Hawaiians is also consistent with prior research [10, 11]. Our prevalence of diabetic disease among Micronesians is similar to that found in an earlier study on gestational diabetes among Micronesians in Hawaii [41]. The high rates of macrosomic infants among Samoans have also been noted in the literature [11, 13].

Although Samoans had the highest prevalence and highest risk of delivering macrosomic infants, they did not have an increased risk of cesarean delivery. Micronesians, on the other hand, had infants with the lowest mean birthweight and the lowest risk of delivering a macrosomic infant yet experienced the highest risk of cesarean delivery.

Anthropometric differences in race/ethnicity may help explain these findings. One would expect Samoans to have an increased risk for cesarean, due to their higher rates of hypertensive and diabetic diseases as well as obesity [42]. According to the Centers for Disease Control and Prevention’s Pregnancy Risk Assessment Monitoring System (PRAMS), Samoans had the highest prevalence of pre-conception overweight and obesity compared to all other ethnic groups studied, followed by Other Pacific Islanders (non-Hawaiians) [43].

This is the largest study using detailed hospital data to examine obstetric outcomes of Pacific Islanders in the United States. Furthermore, this is the largest study examining obstetric outcomes of Micronesians, a rapidly growing population in both the Hawaii and the United States [44]. Because all Hawaii hospitals except for the military hospital were included in this study, our findings are representative of the entire civilian Pacific Islander population in Hawaii.

However, there are several limitations of the study. Only the largest Pacific Islander groups were studied. There is likely heterogeneity within the Other Pacific Islander subgroup and possibly within the other Pacific Islander groups studied here as well. Sample size and hospital classification methods limited studying smaller subgroups separately. The White population in Hawaii (a minority population in Hawaii) may not be representative of the White population in the US [45, 46].

All variables other than age, delivery in rural hospital, birthweight, and insurance status were extracted from ICD-9 codes. Data on some risk factors and potential confounders were not readily available, or were not reliably reported, from discharge ICD-9 codes in the HHIC inpatient database. Thus our models are missing such critical factors as parity, body mass index, tobacco use, education, and early access to prenatal care. These factors, especially parity and body mass index, would have likely impacted results in the multivariate models. Linking our database to birth certificate data or gathering this detailed clinical data from hospital charts would be a fruitful area for future research. Furthermore, this would allow us to study other key perinatal outcomes such as preterm delivery, which is underreported when using ICD-9 codes [47].

This study reveals several important clinical, public health and policy implications. Understanding Pacific Islanders’ specific perinatal risks, clinicians will be able to better counsel and manage their pregnant patients. Because teen birth is associated with poorer medical, social, and economic outcomes for teen mothers and their children, there should be further studies and interventions to assess attitudes towards teen pregnancy, explore the reasons for teen pregnancy, and to assist teenage mothers in all Pacific Islander populations [25, 48–51]. Given that the majority of each Pacific Islander subgroup rely on public insurance or are uninsured, policy should be created to improve access to health care and to improve health care delivery and utilization within the public system.

Pre-existing and gestational hypertensive and diabetic diseases in pregnancy have not only immediate consequence (e.g. congenital anomalies, premature delivery) but also have potential long-term sequelae (renal insufficiency, cardiovascular disease) [34–37]. The disparate rates of these conditions among Pacific Islanders should be investigated further. The barriers and facilitators to prevention and care for these condition among each Pacific Islander population need to be better understood before developing initiatives to educate and support Pacific Islander groups and their health care providers. The impact of the obesity epidemic on these conditions as well as all other perinatal outcomes among Pacific Islander groups should be further studied.

Additional studies and public health interventions should address each population’s specific areas of need. This study identifies many of these for specific Pacific Islander groups. For example, given the higher rates of morbidity for cesarean deliveries compared to vaginal deliveries, as well as societal costs for cesarean deliveries, efforts should be made to decrease the rates of cesarean deliveries for Micronesians. In order to do this, additional studies should be conducted to better understand Micronesians’ increased odds of cesarean deliveries. Likely obesity is playing a factor.

And most importantly, interventions and policy should address the heterogeneity of the Pacific Islander groups and the different settings in which they receive health care. Language and other cultural differences need to be considered. Discrimination likely plays a role in some of these disparities and when present, they should be addressed [52–54].

## Conclusions

Important differences in risk factors for poor obstetric outcomes and in perinatal outcomes among Pacific Islanders exist. Given the heterogeneity within each Pacific Islander subgroup studied, Pacific Islanders should ideally be disaggregated when designing such future research, policy and other public health interventions in order to be most effective.

## Additional file

Additional file 1: **Maternal outcomes.** Diagnoses with respective ICD-9 codes. (DOCX 42 kb)

## Abbreviations

AHRQ: Agency for Healthcare Research and Quality
aOR: Adjusted odds ratios
CI: Confidence intervals
ICD-9: International Classification of Diseases, 9th Revision
HHIC: Hawaii Health Information Corporation
PRAMS: Pregnancy Risk Assessment Monitoring System
US: United States
